# The Influence of Social Exclusion Types on Individuals' Willingness to Word-of-Mouth Recommendation

**DOI:** 10.3389/fpsyg.2022.862003

**Published:** 2022-04-15

**Authors:** Feng Wenting, Wang Lijia, Gao Cuixin

**Affiliations:** ^1^Gemmological Institute, Research Center for Psychological and Health Science, China University of Geosciences (Wuhan), Wuhan, China; ^2^Research Center for Psychological and Health Sciences, Institute of Education, China University of Geosciences (Wuhan), Wuhan, China; ^3^Research Center for Psychological and Health Sciences, Institute of Advanced Studies, China University of Geosciences (Wuhan), Wuhan, China

**Keywords:** social exclusion, being rejected, being ignored, differential needs, word-of-mouth recommendation

## Abstract

As the pace of modern life accelerates, social exclusion occurs more and more frequently in interpersonal interactions. The type of social exclusion can lead to different psychological needs of individuals, and, thus, affects the tendency of word-of-mouth (WOM) recommendation. There are three experiments in this research. Experiment 1 explores the influence of social exclusion types on the willingness of WOM recommendation. The result shows that being rejected increases individuals' willingness to WOM recommendations while being ignored decreases individuals' willingness. Experiment 2 explores the internal psychological mechanism of the influence of social exclusion types on WOM recommendation behavior and proves the mediating role of psychological needs (affiliative-focused needs; power/provocation need). In experiment 3, the moderating effect of product attributes (scarcity/popularity) on the main effect is analyzed. This research is the first to explore the influence of social exclusion types on individuals' willingness to WOM recommendations, which enriches the research on social exclusion in the field of WOM recommendations.

## Introduction

With the rapid development of modern social networks and communication technology, individuals can exchange their experiences and feelings anytime and anywhere and disseminate information about products and services (Raacke and Bonds-Raacke, 2008). This kind of communication among consumers is a word-of-mouth (WOM) recommendation, which enriches interpersonal communication and strengthens information sharing among individuals (Ritson and Elliott, 1999). Besides, it is also an important content of social interpersonal communication. The WOM recommendation has become a new way of social communication with the pronoun of “Anli” (Chinese Internet buzzwords, means recommendation), “Zhongcao” (Same meaning as “Anli”) and “Bacao” (Compared with “Zhongcao,” refers to the implementation of purchase behavior) appearing in interpersonal communication. However, with the change of pace and style of modern life, people are more and more likely to feel social exclusion in their interpersonal communication. Social exclusion is a very bad experience for individuals and an important social factor affecting individuals' psychology and behavior (Williams, 2001). So, how does social exclusion affect the WOM recommendation?

Existing studies on the impact of social exclusion on WOM recommendation present conflicting conclusions. Some studies have shown that the key function of WOM recommendation is to strengthen social relations and to alleviate the adverse experience brought by social exclusion (Berger, 2014). Therefore, the social exclusion will increase individuals' willingness to WOM recommendations (Berger, 2014; Kumar and Kaushal, 2021). Sinha and Lu (2019) found that individuals who experienced social exclusion would improve their brand attitude and willingness to WOM recommendations. Additionally, WOM recommendation reduces interpersonal distance through communication and sharing and makes up for the lack of the sense of belonging caused by social exclusion (Berger, 2014). However, other studies have found that socially excluded individuals are not friendly, and even tend to take aggressive actions to cope with it (Chow et al., 2008). Consequently, they should be less willing to make WOM recommendations to others. Moreover, social exclusion leads to social withdrawal, triggering the desire for solitude and avoiding communication and contact with others (Ren et al., 2016, 2021), and, hence, less likely to make WOM recommendations (Twenge et al., 2007). How does social exclusion affect WOM recommendations? Previous studies have found conflicting findings, which makes it difficult to draw a consistent conclusion from them.

Therefore, based on psychological needs theory (Williams, 2009), the current research firstly takes social exclusion types as the entry point (being rejected/being ignored) to analyze the influence of social exclusion types on individuals' willingness of WOM recommendations. It provides a new perspective to integrate the contradictory viewpoints of previous studies and explains when social exclusion promotes WOM recommendation, which makes up for the limitations of previous research and expands relevant research in the field of social exclusion and WOM recommendation.

There are three experiments in this research. Experiment 1 shows that the type of social exclusion (being rejected/being ignored) could effectively affect individuals' willingness to WOM recommendations. Social exclusion is a phenomenon, in which an individual is rejected, isolated, or ignored by others or groups (Berger, 2014). Being rejected means receiving negative feedback explicitly while being ignored means having no feedback and it is implicit (Molden et al., 2009). Being rejected increases an individual's willingness to WOM recommendations, while being ignored decreases an individual's willingness to it. Experiment 2 explores the mediating role of individual psychological needs (uniqueness/relation) in the relationship between social exclusion types and individuals' willingness to WOM recommendations and verifies the theoretical logic of the main effect. Experiment 3 examines the moderating effect of product attributes (scarcity/popularity) on the main effect and further clarifies the boundary conditions. When the product attribute is popular, being ignored reduces the individual's willingness to WOM recommendations, and being rejected increases the individual's willingness to it. When the product attribute is scarce, the type of social exclusion does not significantly affect individuals' willingness to recommend through WOM.

## Literature Review and Hypothesis Development

### Social Exclusion

With the change of modern lifestyle, social exclusion is increasingly common in everyday life, such as being isolated in chatting, being rejected in job hunting, and being ignored by friends or lovers (Berger, 2014). Social exclusion is a phenomenon, in which an individual is rejected, isolated, or ignored by others or groups (Berger, 2014). Existing studies have found that social exclusion leads to two completely different behavioral responses. Some studies argue that social exclusion increases prosocial behavior. Moreover, social exclusion increases individuals' willingness to cooperate with others and pay for the need for relations (Williams, 2009), and their charitable donation behavior (Lee and Shrum, 2012). However, other studies suggest that social exclusion increases antisocial behavior. For example, social exclusion reduces individuals' willingness to donate and the amount of donations (Lee and Park, 2019) increases their unethical behavior (Kouchaki and Wareham, 2015), and reduces their willingness to help others (Twenge et al., 2007).

Molden et al. (2009) distinguished different types of social exclusion (being rejected/being ignored). Different types of social exclusion have something in common, that is, they are all excluded by specific people or groups. Nevertheless, there are also differences between them. Being rejected means receiving negative feedback; it is explicit while being ignored means having no feedback and it is implicit (Molden et al., 2009). Specifically, being rejected means that individuals receive clear feedback about their bad situation in a relationship or group, and, thus, receive an active rejection. Being ignored refers to the fact that individuals receive more hints of lack of social relations and, thus, are passively ignored (Leary, 2005; Williams, 2007). Studies have shown that different types of social exclusion produce different outcomes. Sinha and Lu (2019) found that being rejected led to the formation of low-level mental structure and activation of specific thinking mode, thus, causing individuals to prefer tangible (visual) compensation. On the other hand, being ignored led to the formation of higher-level mental structure and activation of abstract thinking mode, and in this situation, individuals would prefer intangible (verbal) compensation. Furthermore, Lee and Shrum (2012) suggested that being rejected increased individuals' donation behavior, while being ignored increased individuals' conspicuous consumption behavior.

### The Influence of Social Exclusion Types on Psychological Needs

Social exclusion can threaten four fundamental needs: the need to belong, the need to maintain reasonably high self-esteem, the need to perceive control over one's social environment, and the need to feel recognized for existing and being worthy of attention (Williams, 2009). Belonging and self-esteem become an inclusionary need cluster, such that following social exclusion, individuals behave in ways to either remind themselves of their social connections, or that will improve their chances of belonging (Williams, 2009). Control and existence become a power and provocation need cluster, such that when these needs rise to the top of individuals' priorities, they may be in dominating others and forcing others to recognize their existence (Williams, 2009). Belonging and self-esteem threats may motivate individuals to please others; control and meaningful existence threats might motivate aggressive and provocative responses (Williams, 2007).

Behavioral consequences appear to be split into two general categories: affiliative-focused needs (belonging and self-esteem) and power/provocation needs (control and recognition). If affiliative-focused needs are mostly thwarted, then, ostracized individuals will seek to fortify these needs by thinking, feeling, and behaving in a relatively prosocial manner (Bernstein et al., 2010). People generally have a desire to form and maintain positive interpersonal relationships (Baumeister and Leary, 1995). The affiliative-focused needs encourage individuals to pursue interpersonal relationships more actively and strive to maintain a good public image, and increase their willingness to cooperate in groups (Williams, 2001). Once the power/provocation needs are mostly thwarted, ostracized individuals will attempt to fortify these needs, which, in many instances, may result in controlling, provocative, and even antisocial responses (Warburton et al., 2006; Williams, 2007). Individuals lacking the sense of power/provocation tend to choose unique products to highlight their sense of existence (Wan et al., 2014). Hence, different types of social exclusion threaten different psychological needs, and then, lead to different behaviors of individuals (Lee and Shrum, 2012; Wesselmann et al., 2015).

This research purposes that social exclusion types (being rejected/being ignored) affect the psychological needs of individuals. Specifically, being rejected threatens individuals' sense of belonging, and the lack of the sense of belonging activates affiliative-focused needs. Being rejected means that an individual receives clear feedback about his or her bad status in social relations (Leary, 2005). That is to say, being rejected denies an individual's qualification as a member of a group. An individual's inability to possess in-group membership means being excluded from the group, which threatens his or her sense of belonging to the group (Baumeister and Leary, 1995). When the sense of belonging of an individual is threatened, he or she will have a strong desire to rebuild the connection with others, for example, to participate in group activities more actively, or even to purchase group exclusive items to seek recognition (Wan et al., 2014). When the individual's sense of belonging is threatened, the dominant need of him or her is the affiliative-focused needs (Lee and Shrum, 2012). Thus, being rejected threatens individuals' sense of belonging, thereby activating affiliative-focused needs.

However, being ignored threatens individuals' sense of meaningful existence, thereby activating the power/provocation need. Specifically, unlike explicitly being rejected, being ignored is unilateral, and individuals who are ignored do not know the reason (Williams, 2009). Being ignored is considered as the death of social significance (Williams, 2007; Hales, 2018), which causes individuals to be threatened to lose their belief in their meaningful existence (Williams, 2007). That is, when individuals' meaningful existence is threatened, they will feel a sense of being unimportant and being unnoticed, which makes them feel insignificant. Hence, the absence of a meaningful presence enhances the power/provocation needed to obtain attention (Warburton and Williams, 2005). Moreover, gaining attention can restore social visibility and confirm the existence of individuals, and seeking uniqueness is an important way to gain attention and restore the power/provocation need (Lee and Shrum, 2012). When individuals' meaningful existence is threatened, their dominant need is the power/provocation need. Therefore, being ignored threatens individuals' sense of meaningful existence, and they'll activate the power/provocation need.

### The Influence of Social Exclusion Types on Willingness to WOM Recommendation

The WOM recommendation is the informal communication between consumers on the ownership and the usefulness of a certain product, as well as the services provided by the seller of the product (Westbrook, 1987). The WOM is an important content of social interpersonal communication. It enriches the content of interpersonal communication, strengthens the information sharing between individuals, and enhances the connection between each other. The WOM recommendation has five social functions: impression management, emotion regulation, information acquisition, persuasion, and social bonding (Berger, 2014). Individuals can achieve self-promotion and identity display through WOM recommendations (Packard and Wooten, 2013). As a result, people often make WOM recommendations to others to show that they are “professional” and, at the same time, to project a good image of being helpful. The emotion regulation function of WOM recommendation allows individuals to express and channel their emotions to reduce maladjusted feelings through sharing when they experience adversity (Dichter, 1966). The information acquisition function of WOM recommendation is reflected in those individuals, who obtain relevant information about products or things they are interested in through WOM recommendation (Sweeney et al., 2012; Berger, 2014). The persuasion function of WOM recommendation is reflected in the sales and social situations, where salesmen persuade customers to buy, or friends persuade each other to buy a certain product or carry out a joint activity (Roskos-Ewoldsen, 1997). Through WOM recommendation, individuals communicate with others by emotional communication, impression management, information acquisition, and persuasion to strengthen the common ground and finally establish and consolidate the connection with others (Berger, 2014). Thus, WOM recommendation is an indispensable component of social relationships. In addition, social exclusion, at present, is becoming more and more common in a relationship. Therefore, this research takes the type of social exclusion as the starting point to analyze the influence of social exclusion types (being rejected/being ignored) on individuals' willingness to WOM recommendations to enrich the relevant research of social exclusion in the social field.

In this research, the type of social exclusion (being rejected/being ignored) would influence the individuals' psychological needs (affiliative-focused needs, power/provocation need), thus, prompting them to produce WOM recommendations (Lee and Shrum, 2012). Specifically, when individuals are rejected, their sense of social belonging is threatened and their affiliative-focused needs are enhanced, and, thus, their willingness of WOM recommendation increases. Being rejected means that individuals are explicitly excluded from the group, resulting in their loss of social belonging. When individuals' sense of belonging is threatened, they will recover this by rebuilding social relations (Lee and Shrum, 2012), and WOM recommendation is an important way to rebuild it. Existing studies have shown that WOM recommendations can promote the social connection between people (Sun et al., 2006; Berger, 2014). WOM recommendations can also establish contact with strangers and can maintain contact with acquaintances as well (Chen, 2017). Similarly, this kind of recommendation is the information sharing between individuals, which makes different individuals have common ground and strengthens the social bonds between them (Ritson and Elliott, 1999). Prior research also suggests that talking about popular ads gives teenagers a common topic of conversation, which, in itself, is a social currency, allowing them to integrate with their peers and show that they belong to a group (Ritson and Elliott, 1999). Thus, individuals who are rejected can strengthen their WOM recommendations to rebuild social relations and restore affiliative-focused needs.

However, when individuals are ignored, their sense of meaningful existence is threatened, thus, enhancing their power/provocation need and reducing their willingness to WOM recommendation. Specifically, when individuals are ignored, they cannot communicate directly with the group that ignores them, let alone be the determining reason for being ignored (Warburton and Williams, 2005; Williams, 2009). At this point, individuals tend to get attention by highlighting their uniqueness to restore their sense of meaningful existence and meet the power/provocation need (Wan et al., 2014). Uniqueness refers to the tendency to define oneself as a distinction from the members of its reference group (Bloch, 1995). The need for uniqueness leads to a high desire to own unique products (Simonson and Nowlis, 2000). However, once the public became aware that these products are already purchased, owned, or used, the novelty products eventually lose their uniqueness (Granovetter and Soong, 1986). Thus, uniqueness drives individuals to fear becoming like others, thus, threatening their uniqueness. However, sharing information with others can reduce one's uniqueness (Ritson and Elliott, 1999). Consequently, ignored individuals are less inclined to engage in WOM recommendations due to activation of the power/provocation need.

H1: Social exclusion types (being rejected/being ignored) can significantly affect individuals' willingness to WOM recommendations. Being rejected can lead to a significantly higher willingness for WOM recommendations than being ignored.

H2: Psychological needs (affiliative-focused needs, power/provocation need) of individuals mediate the relationship between the social exclusion types (being rejected/being ignored) and willingness of WOM recommendations.

### The Moderating Effect of Product Attributes: Scarcity and Popularity

Scarcity and popularity are common cues of product attributes. Scarcity cues are mostly used for limited-edition products, while popularity cues are mostly used for popular products (Hélène et al., 2013). Scarce products do not have a large quantity of supply and are generally limited and unique, while popular products have a large number of shipments and are generally very common (Gierl et al., 2008). Product attributes have very strong symbolic significance and can meet different psychological needs of individuals, and they are also important factors affecting individuals' behavior and decision-making (Snyder and Fromkin, 1980; Çelen and Kariv, 2004).

Product attributes (scarcity/popularity) can bring different social symbolic meanings to individuals (Robinson et al., 2016). Scarce products can convey individual uniqueness, and individuals can highlight their uniqueness in the crowd by purchasing scarce products (Parker and Lehmann, 2011). Therefore, individuals with unique needs prefer scarce products (Fromkin, 1970). Popular products, however, imply group relationships. The psychological reason behind the popularity is that individuals desire to become a part of a group and establish relationships with others in the group by consuming the same products (Jeong and Kwon, 2012). Relatively, when individuals imitate others to purchase popular products, they will think that they are more acceptable to others (Ha et al., 2016).

In this context, product attributes (scarcity/popularity) can effectively influence the relationship between social exclusion types (being rejected/being ignored) and WOM recommendation behavior. Specifically, when the product is popular, to meet the affiliative-focused needs, individuals who are rejected can better establish and maintain contact with others by recommending the popular product (Leibenstein, 1950). On this condition, individuals who are rejected are more likely to make WOM recommendations. However, for individuals who are ignored, being ignored activates their power/provocation need, and recommending a popular product will highlight the popularity of their taste and further reduce their uniqueness (Granovetter and Soong, 1986). However, power/provocation need leads to keeping uniqueness. Hence, individuals who are ignored are not inclined to make WOM recommendations. When the product is scarce, its audience scope is small, and its uniqueness is not easy to be widely accepted by social groups. It is difficult for individuals to obtain common ground or establish group relationships through recommending such products, and, thus, the social risk of recommending them by WOM is very high (DeSarbo et al., 2002). Hence, individuals who are rejected are less inclined to recommend such products by WOM. For individuals who are ignored, owning scarce products is an important way to meet their needs for uniqueness (Parker and Lehmann, 2011). If others have the same scarce products as them, their uniqueness will be threatened. Therefore, to meet their power/provocation need, individuals who are ignored are not inclined to recommend scarce products by WOM.

H3: Product attributes (scarcity/popularity) significantly moderate the relationship between social exclusion types and WOM recommendations. When the product is scarce, rejected individuals have affiliative-focused needs and are less likely to recommend by WOM. Meanwhile, neglected individuals are also not inclined to recommend by WOM for scarce products to satisfy their power/provocation need. When the product is popular, rejected individuals are more likely to recommend it by WOM to satisfy the affiliative-focused needs, while neglected individuals have power/provocation needs and are less likely to recommend through WOM.

## Methods

### Study 1

The purpose of experiment 1 is to test that social exclusion types (being rejected/being ignored) can significantly affect individuals' willingness to WOM recommendations.

#### Participants and Design

Based on the calculation method adopted by Cohen (1977) (the effect size *f* = 0.25 and the expected power = 0.80), the researcher determined sample sizes of more than 159 by G^*^Power 3.1 software. Therefore, experiment 1 recruited 180 participants, who are mainly students from a university, including undergraduates, postgraduates, and doctoral students. The final sample was *N* = 171 (*M*_*age*_ = 21.41*, SD*_*age*_ = 2.56, age range: 17–29, female 52.78%). Participants were randomly assigned to three experimental designs (being ignored, being rejected, or control), and each group was (n _*beingrejected*_ = 57, n _*beingignored*_ = 56, and n _*control*_ = 58).

#### Stimuli and Procedure

Participants were told that this experiment aimed at developing a psychological counseling technique for college students. Participants were randomly assigned to being ignored, being rejected, or control experimental conditions. Researchers used recalling and writing tasks to manipulate social exclusion types (Molden et al., 2009). The task asked participants in social exclusion (being ignored/being rejected) groups to recall an incident, in which they had been ignored or rejected, and then, write it down in 5 min. In the “being ignored group,” participants were asked to “write down a moment when you felt strongly ignored in some way. At that time, you were obviously ignored, but no one actually said they didn't want or like you.” In the “being rejected group,” participants were asked to “write down a moment when you felt strongly rejected in some way. At that time, you were obviously rejected and clearly told that you were not accepted because they did not want or like you.” In the control group, participants were asked to recall and write down life events when they drove or walked to the supermarket. The researcher checked the writing content in the task. When the content was consistent with the recall task, it was coded as “0”; when the content was inconsistent with the recall task, it was coded as “1.” Then, all participants were given a picture of a task reward (a picture of a T-shirt) and then were asked to complete a series of questionnaires, including a report on their willingness to recommend the item by WOM: “If you owned this product, would you recommend this item to your friends?” (7-point scale, 1: *Very Unwilling*; 7: *Very Willing*) (Cheema and Kaikati, 2010), the degree of being ignored and being rejected that they perceived (7-point scale, 1: *Very Low*; 7: *Very High*) (Molden et al., 2009), and some items, such as personal interests and hobbies and comments on t-shirts. Then, they reported whether their willingness of WOM recommendations was based on their past shopping experience and guessed the purpose of this experiment.

#### Results and Discussion

##### Manipulation Check

All the participants reported the consistent contents in the task, 9 participants' willingness of recommendation depended on past shopping experience, and no participants guessed the real purpose of the experiment. There was a significant difference in the feeling of being rejected among the three groups (*F* = *117.57, P*< *0.001, ES* = *0.58*). Participants in the being rejected group felt significantly more rejected than those in the control group (*M*_*beingrejected*_ = *5.37, SD* = *1.05, M*_*control*_ = *3.17, SD* = *0.80, t* = *13.51, df* = *168, p*< *0.001, d* = *2.36*) and being ignored group (*M*_*beingignored*_ = *3.23, SD* = *0.74, t* = *13.03, df* = *168, p*< *0.001, d* = *2.36*). The ignored group and the control group reported no significant distinction on being rejected (*t* = *0.37, df* = *168, p* = *0.715, d* = *0.08*). There was a significant difference in the feeling of being ignored among the three groups (*F* = *201.97, p*< *0.001, ES* = *0.71*). Participants in the being ignored group felt significantly more ignored than those in the being rejected group (*M*_*beingignored*_ = *5.54, SD* = *0.81, M*_*beingrejected*_ = *3.16, SD* = *0.59, t* = *16.95, df* = *168, p*< *0.001, d* = *3.36*) and control group (*M*_*control*_ = *3.03, SD* = *0.82, t* = *17.90, df* = *168, p*< *0.001, d* = *3.08*). The rejected group and the control group reported no significant distinction on being ignored (*t* = *0.89, df* = *168, p* = *0.376, d* = *0.18*). The results showed that the experiment manipulation effectively influenced the majority of participants.

##### Willingness to WOM Recommendation

The results showed that there was a significant difference in the willingness to WOM recommendation among the three groups (*F* = *58.86, p*< *0.001, ES* = *0.41*). The willingness to WOM recommendation in the being rejected group was significantly higher than that in the control group (*M*_*beingrejected*_ = *5.25, SD* = *0.83, M*_*control*_ = *4.12, SD* = *0.94, t* = *6.65, df* =*168, p*< *0.001, d* = *1.27*) and being ignored group (*M*_*beingrejected*_ = *5.25, SD* = *0.83, M*_*beingignored*_ = *3.41, SD* = *0.95, t* = *10.75, df* =*168, p*< *0.001, d* = *2.06*). At the same time, the WOM willingness of the being ignored group was significantly lower than that of the control group (*M*_*beingignored*_ = *3.41, SD* = *0.95, M*_*control*_ = *4.12, SD* = *0.94, t* = *4.18, df* = *168, p*< *0.001, d* = *0.75*). The results of experiment 1 verified hypothesis 1, as shown in Supplementary Figure 1. Experiment 1 verified hypothesis 1, that social exclusion types (being rejected/being ignored) can significantly affect the willingness of WOM recommendation. Being rejected will increase the willingness to WOM recommendations while being ignored will reduce the willingness to WOM recommendations.

### Study 2

The purpose of experiment 2 is to test H2, psychological needs (affiliative-focused needs; power/provocation need) mediate the relationship between social exclusion types (being rejected/being ignored), and willingness to WOM recommendation.

#### Participants and Design

Based on the calculation method adopted by Cohen (1977) (the effect size *d* = 0.5 and the expected power = 0.80), the researcher determined sample sizes of more than 128 by G^*^Power 3.1 software. Therefore, experiment 2 recruited 140 participants, who are mainly students from a university, including undergraduates, postgraduates, and doctoral students. The final sample was *N* = 133 (*M*_*age*_ = 21.99*, SD*_*age*_ = 2.10, age range: 18–31, female 45.11%). The participants were randomly assigned to two experimental designs (being ignored, being rejected), and each group was (n _*beingrejected*_ = 66, n _*beingignored*_ = 67).

#### Stimuli and Procedure

Experiment 2 conducted the social rejection experiment of Molden et al. (2009). Participants were told that this experiment was about online social interaction. They would discuss two randomly selected topics with two other participants in an online chat room. In effect, the other two participants were false participants played by researchers. In the experiment, real participants would receive email prompts and always be the first to speak. The two false participants would cooperate to reject or ignore the real participant with the preset dialogue content. In the “being ignored situation,” no matter what real participants sent out, they would be ignored by false participants, and the two false participants would have a dialogue in pairs. In the “being rejected condition,” no matter what real participants sent out, they would receive negation and refutation from two false participants. When the conversation lasted about 10 min, all participants received a message indicating that the conversation task was over. To control the psychological distance, all participants were asked to complete the following rating tasks as bystanders. Then, participants were asked to complete a series of questionnaires about their psychological needs (affiliative-focused needs; power/provocation need, 7-point scale) (Williams, 2009) and their perceived degree of being ignored and rejected (7-point scale, 1: *Very Low*; 7: *Very High*) (Molden et al., 2009). Finally, participants were told they would be given a hat as a gift (presented with a picture of the hat), and then, they should report their willingness to recommend the item by WOM: “Would you recommend this hat to your friends?” (7-point scale, 1: *Very Unwilling*; 7: *Very Willing*) (Cheema and Kaikati, 2010), as well as other items. The emotion dimension scale of Hagtvedt (2011) was used to measure their emotional state. Finally, participants reported their psychological distance (1: *Very Close*; 7: *Very Far*) (Niu et al., 2010), whether their willingness of WOM recommendation was based on their past shopping experience and guessed the purpose of this experiment.

#### Results and Discussion

##### Manipulation Check

The willingness to recommend of the 7 participants depended on past shopping experience, and no participants guessed the real purpose of the experiment. There was no significant difference in emotional state between the two groups (*M*_*beingignored*_ = *2.79, SD* = *0.84, M*_*beingrejected*_ = *2.80, SD* = *0.83, t* = *0.08, df* = *131, p* = *0.934, d* = *0.01*). There was no significant difference in psychological distance between the two groups (*M*_*beingignored*_ = *5.18, SD* = *0.85, M*_*beingrejected*_ = *5.21, SD* = *0.89, t* = *0.22, df* = *131, p* = *0.827, d* = *0.03*). Participants in the being rejected group felt significantly more rejected than those in the being ignored group (*M*_*beingrejected*_ = *5.44, SD* = *0.95, M*_*beingignored*_ = *3.67, SD* = *0.88, t* = *11.17, df* = *131, p*< *0.001, d* =*1.93*). Participants in the “being ignored group” felt significantly more ignored than those in the being rejected group (*M*_*beingignored*_ = *5.16, SD* = *0.91, M*_*beingrejected*_ = *3.24, SD* = *0.90, t* = *12.24, df* = *131, p*< *0.001, d* = *2.12*). The results showed that the experiment manipulation effectively influenced the majority of participants.

##### Psychological Needs

Participants in the being rejected group report significantly higher affiliative-focused needs than those in the being ignored group (*M*_*beingrejected*_ = *5.22, SD* = *1.01, M*_*beingignored*_ = *3.61, SD* = *0.92, t* = *9.58, df* = *131, p*< *0.001, d* = *1.67*). Participants in the being ignored group reported significantly higher power/provocation need than those in the being rejected group (*M*_*beingignored*_ = *5.42, SD* = *0.88, M*_*beingrejected*_ = *3.89, SD* = *1.05, t* = *9.15, df* = *131, p*<*0.001, d* =*1.58*). The results showed that the need for power/provocation in the being ignored group was significantly higher than that in the being rejected group, while the affiliative-focused need in the being rejected group was significantly higher than that in the being ignored group.

##### The Willingness to WOM Recommendation

There was a significant difference in the willingness to WOM recommendation between the two groups. The willingness to WOM recommendation in the being ignored group was significantly lower than that in the being rejected group (*M*_*beingignored*_ = *3.64, SD* = *0.90, M*_*beingrejected*_ = *5.23, SD* = *1.06, t* = *9.29, df* = *131, p*< *0.001, d* = *1.62*), again verifying the main effect of the research.

##### The Analysis of the Mediating Effect

To further test the relationship among social exclusion types, psychological needs (affiliative-focused needs or power/provocation need), and WOM recommendation, this research analyzed the mediating effect of psychological needs. A bootstrapping analysis (PROCESS Model 4, Hayes, 2013, with 10,000 bootstrapping resamples) was used to analyze the mediating role of psychological needs. The results showed that affiliative-focused needs mediated the influence of social exclusion types on the willingness to WOM recommendation (95% confidence interval β =1.60; CI = 1.26–1.94). Power/provocation need also mediated the influence of social exclusion types on the willingness to WOM recommendation (95% confidence interval β = 1.54; CI = 1.21–1.89). See Supplementary Figure 2 for details.

Experiment 2 verified H2 and analyzed the mediating role of psychological needs (affiliative-focused needs or power/provocation need). It indicated that psychological needs (affiliative-focused needs or power/provocation need) mediated the relationship between social exclusion types and willingness to WOM recommendation, and proved the theoretical logic of the main effect.

### Study 3

The purpose of experiment 3 is to test H3, the moderating effect of product attributes (scarcity/popularity) on the relationship between social exclusion and individuals' willingness to WOM recommendations.

#### Participants and Design

Based on the calculation method adopted by Cohen (1977) (the Effect size *f* = 0.25 and the expected Power = 0.80), the researcher determined sample sizes of more than 179 by G^*^Power 3.1 software. Therefore, experiment 3 recruited 200 participants. The final sample was *N* = 185 (*M*_*age*_ = 21.74*, SD*_*age*_ = 2.26, age range: 18–29, female 41.62%). Participants were randomly assigned to 2 (social exclusion types: being ignored/being rejected) × 2(product attributes: scarcity/popularity) experimental design, and each group was (*n*_*rejectpopularity*_ = *47, n*_*rejectscarcity*_ = *47, n*_*ignorepopularity*_ = *46, n*_*ignorescarcity*_ = *45*).

#### Stimuli and Procedure

Experiment 3 adopted the manipulation method of product attributes of Wu and Lee (2016). All participants were prompted to buy coffee cups from online retailers. The manipulation of scarce products was described as “Product A is an annual special limited edition,” and the manipulation of popular products was described as “75% of consumers bought this product B after viewing this site.” The researcher recruited 60 participants online for pre-test (*M*_*age*_ = 22.55*, SD*_*age*_ = 2.48, age range: 20–29, female 58.33%). All participants were randomly assigned to the scarcity group or the popularity group, and products were presented to participants correspondingly. Then the participants were asked to rate the product attributes (7-point scale, 1: *Very Scarce*; 7: *Very Popular*) (Wu and Lee, 2016). The results showed that participants in the scarcity group rated product attributes significantly lower than those in the popularity group (*M*_*scarcity*_ =*3.13, SD* = *0.82, M*_*popularity*_ =*5.77, SD* = *0.90, t* =*11.87, df* =*58, p*< *0.001, d* = *3.07*), verifying the effectiveness of product attributes manipulation.

The manipulation of social exclusion types (being ignored/being rejected) was similar to experiment 2. To control psychological distance, all participants were asked to complete the following rating tasks as bystanders. Participants were then told that they would receive a coffee mug as a gift (presented with a picture of the mug). Participants in the scarcity group were told that the mug was a special annual limited edition, while participants in the popularity group were told that 75% of consumers bought this mug after viewing this site. After that, all participants completed a series of questionnaires, including the degree of perceived “being ignored” and “being rejected” (7-point scale, 1: *Very Low*; 7: *Very High*) (Molden et al., 2009), their psychological needs (affiliative-focused needs; power/provocation need,7-point scale) (Williams, 2009), their willingness to recommend the coffee cup by WOM: “Would you recommend this cup to others?” (7-point scale, 1: *Very Unwilling*; 7: *Very Willing*) (Cheema and Kaikati, 2010), and other items. Finally, the participants rated the product attributes (7-point scale, 1: *Very Scarce*; 7: *Very Popular*) (Wu and Lee, 2016), and completed the emotion state measurement. Moreover, participants reported their psychological distance, whether their willingness to WOM recommendation was based on their past shopping experience and guessed the purpose of this experiment.

#### Results and Discussion

##### Manipulation Check

The willingness to recommend of 15 participants depended on past shopping experience, and no participants guessed the real purpose of the experiment. There was no significant difference in emotional state between the two groups (*M*_*beingignored*_ = *2.89, SD* = *0.78, M*_*beingrejected*_ = *2.79, SD* = *0.73, t* = *0.93, df* = *183, p* = *0.356, d* = *0.13*). There was no significant difference in psychological distance between the two groups (*M*_*beingignored*_ = *5.26, SD* = *0.99, M*_*beingrejected*_ = *5.06, SD* = *1.12, t* = *1.28, df* = *183, p* = *0.20, d* = *0.19*). Participants in the “being rejected group” felt significantly more rejected than those in the being ignored group (*M*_*beingrejected*_ = *5.43, SD* = *0.97, M*_*beingignored*_ = *3.38, SD* = *0.96, t* = *14.38, df* = *183, p*< *0.001, d* = *2.12*). Participants in the being ignored group felt significantly more ignored than those in the being rejected group (*M*_*beingignored*_ = *5.46, SD* = *0.97, M*_*beingrejected*_ = *3.44, SD* = *1, t* = *13.98, df* = *183, p*< *0.001, d* = *2.05*). In addition, participants in the scarcity group rated the product attributes significantly lower than those in the popularity group (*M*_*scarcity*_ = *3.27, SD* = *0.92, M*_*popularity*_ = *5.55, SD* = *0.89, t* = *17.15, df* = *183, p*< *0.001, d* = *2.52*). The results showed that the experiment manipulation effectively influenced the majority of participants.

##### Psychological Needs

The psychological needs of the two groups were significantly different. Participants in the “being rejected group” reported a significantly higher affiliative-focused need than those in the being ignored group (*M*_*beingrejected*_ = *5.59, SD* = *0.87, M*_*beingignored*_ = *3.65, SD* = *0.91, t* = *14.83, df* = *183, p*< *0.001, d* = *2.18*). Participants in the ‘being ignored group' reported a significantly higher power/provocation need than those in the being rejected group (*M*_*beingignored*_ = *5.55, SD* = *0.87, M*_*beingrejected*_ = *3.52, SD* = *0.97, t* = *15, df* = *183, p*< *0.001, d* = *2.20*). The results showed that the “being ignored group” had more significant need for power/provocation need, while the “being rejected group” had more significant affiliative-focused need.

##### Willingness to WOM Recommendation

The results showed that the interaction between social exclusion types and product attributes significantly affected willingness to WOM recommendation (*F* = *88.44, p*< *0.001, ES* = *0.25*). When the product was a popular product, participants in the “being rejected group” were significantly and more likely to recommend coffee cups by WOM than those in the being ignored group (*M*_*beingrejected*_ = *5.36, SD* = *0.94, M*_*beingignored*_ = *3.33, SD* = *0.82, t* = *11.12, df* = *91, p*< *0.001, d* = *2.30*). However, when the product was unique, there was no significant difference between the two groups in willingness to WOM recommendation (*M*_*beingrejected*_ = *3.17, SD* = *0.94; M*_*beingignored*_ = *3.53, SD* = *0.94, t* = *1.85, df* = *90, p* = *0.068, d* = *0.38*).

Moderated mediation analysis: The data were submitted to a moderated mediation analysis (using the macro-PROCESS, model 15, with 10,000 bootstrapping resamples; see Hayes, 2013). The independent variable (X) was a dummy variable representing the two experimental conditions (being ignored/being rejected). The moderator (V) was a dummy variable representing the two conditions (product attributes: scarcity/popularity). The dependent variable (Y) was the willingness of WOM recommendation. The mediator was the psychological needs (affiliative-focused needs or power/provocation needs) (M).

When the mediator was affiliative-focused needs, the results revealed that the moderating effect of product attributes was significant (95% confidence interval β = 1.94; CI = 1.34–2.64). Specifically, under popularity product conditions, the indirect effect of social exclusion type on the willingness to WOM recommendation was significant (95% confidence interval β = 1.98; CI = 1.71–2.26). Under scarce product condition, no distinctions appeared between the two groups (95% confidence interval β = 0.04; CI = −0.56 to 0.61). See Supplementary Figure 3 for details.

When the mediator was power/provocation need, the results revealed that the moderating effect of product attributes was also significant (95% confidence interval β = 1.94; CI = 1.31–2.64). Specifically, under popularity product conditions, the indirect effect of social exclusion type on the willingness to WOM recommendation was significant (95% confidence interval β = 1.94; CI = 1.68–2.20). Under scarce product condition, no distinctions appeared between the two groups (95% confidence interval β = 0.003; CI = −0.62 to 0.62). See Supplementary Figure 3 for details.

Experiment 3 proved H3 and analyzed the moderating effect of product attributes (scarcity/popularity) on the relationship between social exclusion types and individuals' willingness to WOM recommendations. The results showed that only when the product was popular, social exclusion types could effectively affect individuals' willingness to WOM recommendations.

## General Discussion

### Conclusion

There were three experiments in this research. Experiment 1 showed that social exclusion types (being rejected/being ignored) could effectively affect individuals' willingness to WOM recommendations. That is, being ignored would reduce individuals' willingness to WOM recommendations, and being rejected would increase individuals' willingness to it, verifying the main effect of the research. Experiment 2 demonstrated the mediating role of psychological needs (affiliative-focused needs; power/provocation need) between social exclusion types and individuals' willingness to WOM recommendations, proved the theoretical logic of the main effect, and constructed a complete internal mechanism model. Being rejected (being ignored) activates individuals' affiliative-focused needs (power/provocation need) and, thus, increases (decreases) their willingness to WOM recommendations. Experiment 3 clarified the moderating effect of product attributes (scarcity/popularity) on the main effect. For popular products, social exclusion types (being rejected/being ignored) can effectively affect individuals' willingness of WOM recommendation; However, for scarce products, social exclusion types (being rejected/being ignored) do not significantly affect individuals' willingness to WOM recommendation.

### Theoretical Contributions

The theoretical contributions of this research are mainly reflected in the following aspects: First, this research enriches relevant research in the field of social exclusion. Existing relevant studies have presented conflicting conclusions. Some studies believe that WOM recommendation can strengthen social relationships, and make up for an individual's lack of sense of belonging caused by social exclusion. Therefore, social exclusion can promote individuals' WOM recommendations (Berger, 2014). Other studies suggest that social exclusion leads to antisocial or social withdrawal behaviors and less willingness to make WOM recommendations (Twenge et al., 2007; Chow et al., 2008). How will social exclusion affect WOM? Previous studies cannot draw a consistent conclusion. The current research takes social exclusion types as the entry point to explore the impact of social exclusion types (being rejected/being ignored) on the willingness of WOM recommendation, which provides a new perspective to integrate the contradictory viewpoints of previous studies. It helps to deepen theoretical development, as well as expand relevant research in the field of social exclusion and WOM recommendation.

Second, based on psychological needs theory, this research clarifies the mediating mechanism of the influence of social exclusion types (being rejected/being ignored) on WOM recommendations. Different types of social exclusion (being rejected/being ignored) threaten different psychological needs of individuals (affiliative-focused needs, power/provocation need), resulting in different behavioral outcomes. This research analyzes how social exclusion types (being rejected, being ignored) affect individuals' WOM recommendation through individuals' psychological needs (affiliative-focused needs, power/provocation need). Besides, this research examines the threat of being rejected (being ignored) to the sense of belonging (meaningful existence), which activates individuals' affiliative-focused needs (power/provocation need), to increase (reduce) their WOM recommendations. All in all, this research constructs a complete internal mechanism model, greatly enriching the research in the field of WOM recommendation.

Third, this research introduces product attributes (scarcity/popularity), focuses on their moderating effect on the relationship between social exclusion types and individuals' WOM recommendation, and expands the literature on product attributes. Previous studies on product attributes (scarcity/popularity) focused on the influence of product scarcity and popularity on consumers and the market (Wu and Lee, 2016; Shi et al., 2020), and few studies explored its impact on consumers' WOM recommendations. Taking consumer behavior as the research context, this research first identifies individuals' preference for product attributes under the condition of social exclusion, and clearly defines the moderating role of product attributes. This research proposes that due to the rejected individuals' affiliative-focused needs and the neglected individuals' power/provocation needs, both are not inclined to recommend scarce products by WOM. However, when the product is popular, the rejected individuals can meet the affiliative-focused needs through it, so they are more likely to recommend by WOM, while the neglected individuals need power/provocation and are unlikely to provide WOM recommendations. This research demonstrates the moderating effect of product attributes, establishes clear boundary conditions for the main effect, and constructs a clear framework in the theoretical and applied fields. It is conducive in helping socially excluded individuals select appropriate products through WOM recommendations. That is, for individuals who are rejected, it is more appropriate to recommend popular products. For individuals who are ignored, WOM recommendations should be carefully used to cope with social exclusion and avoid causing psychological discomfort.

### Future Research

This research is the first to explain the influence of social exclusion types (being rejected/being ignored) on individuals' willingness of WOM recommendations from the perspective of psychological needs theory. Future research can further explore whether other mediating variables affect the relationship between social exclusion and individuals' willingness of WOM recommendations. Studies have shown that when suffering from social exclusion, individuals who are extremely eager to rebuild social relations will increase their spending on subordinate services (Baumeister and Leary, 1995) and become more obedient to group opinions. However, this research only studies from the perspective of individuals' WOM recommendations and does not discuss the influence of social exclusion on individuals' acceptance of WOM recommendations. Therefore, future research can focus on the influence of social exclusion on WOM acceptance behavior and the corresponding internal mechanism, which echoes the hot social topic of “payola.” Finally, social exclusion can happen at any time and place. Social exclusion at different times and places may have different effects on individuals' WOM recommendations. Subsequent research can further explore the influence of temporal and spatial background differences on WOM recommendation. This research also explored the moderating effect of product attributes (scarcity/popularity) on the relationship between social exclusion types and individuals' willingness to WOM recommendations. There are many other categories of product attributes, such as public products/private products and hedonic products/utilitarian products. Many other moderating variables can be further explored in future research.

## Data Availability Statement

The original contributions presented in the study are included in the article/Supplementary Material, further inquiries can be directed to the corresponding author/s.

## Ethics Statement

The studies involving human participants were reviewed and approved by the Ethics Committee of Research Center for Psychological and Health Sciences, China University of Geosciences. The patients/participants provided their written informed consent to participate in this study.

## Author Contributions

FW was responsible for the logical reasoning of the research topic. WL was responsible for experimental materials and data. GC was responsible for collecting literature. All authors contributed to the article and approved the submitted version.

## Funding

The authors acknowledge financial support from the National Nature Science Foundation of China (Grant Nos. 72172107, 71702177, and 71532011).

## Conflict of Interest

The authors declare that the research was conducted in the absence of any commercial or financial relationships that could be construed as a potential conflict of interest.

## Publisher's Note

All claims expressed in this article are solely those of the authors and do not necessarily represent those of their affiliated organizations, or those of the publisher, the editors and the reviewers. Any product that may be evaluated in this article, or claim that may be made by its manufacturer, is not guaranteed or endorsed by the publisher.
