# Supplementary material for: The Influence of Social Exclusion Types on Individuals' Willingness to Word-of-Mouth Recommendation
Source: Front Psychol. 2022 Apr 15;13:862003. doi: 10.3389/fpsyg.2022.862003 (PMC9051444; doi:10.3389/fpsyg.2022.862003)
Supplement: Supplementary file 4 [file Table_4.DOCX]

***Supplementary Material***

# Figures

# Figure 1

Results of experiment 1

# Figure 2

social exclusion types

willingness of WOM recommendation recommendation

affiliative-focused needs

Indirect effcet：*β* = 1.60; *CI* = 1.26 to 1.94

Direct effcet：*β* = -0.02; *CI* = -0.11 to 0.08

*β* = 1.61; *CI* = 1.28 to 1.94

*β* = 0.99; *CI* = 0.96 to 1.03

social exclusion types

willingness of WOM recommendation recommendation

power/provocation need

Indirect effcet：*β* = 1.54; *CI* = 1.21 to 1.89

Direct effcet：*β* = 0.04; *CI* = -0.02 to 0.11

*β* = -1.53; *CI* = -1.86 to -1.20

*β* = -1.01; *CI* = -1.03 to -0.98

The SPSS results for the mediating effect of affiliative-focused need

Run MATRIX procedure:

***************** PROCESS Procedure for SPSS Release 2.13 ***************

Written by Andrew F. Hayes, Ph.D. www.afhayes.com

Documentation available in Hayes (2013). www.guilford.com/p/hayes3

**************************************************************************

Model = 4

Y = recom

X = group

M = relation

Sample size

133

**************************************************************************

Outcome: relation

Model Summary

R R-sq MSE F df1 df2 p

.6419 .4120 .9402 91.7808 1.0000 131.0000 .0000

Model

coeff se t p LLCI ULCI

constant 3.6070 .1185 30.4493 .0000 3.3727 3.8414

group 1.6110 .1682 9.5802 .0000 1.2784 1.9437

**************************************************************************

Outcome: recom

Model Summary

R R-sq MSE F df1 df2 p

.9871 .9743 .0417 2461.4313 2.0000 130.0000 .0000

Model

coeff se t p LLCI ULCI

constant .0581 .0709 .8198 .4138 -.0821 .1983

relation .9935 .0184 54.0124 .0000 .9571 1.0299

group -.0151 .0462 -.3273 .7440 -.1065 .0762

******************** DIRECT AND INDIRECT EFFECTS *************************

Direct effect of X on Y

Effect SE t p LLCI ULCI

-.0151 .0462 -.3273 .7440 -.1065 .0762

Indirect effect of X on Y

Effect Boot SE BootLLCI BootULCI

relation 1.6006 .1729 1.2619 1.9395

******************** ANALYSIS NOTES AND WARNINGS *************************

Number of bootstrap samples for bias corrected bootstrap confidence intervals:

10000

Level of confidence for all confidence intervals in output:

95.00

NOTE: Some cases were deleted due to missing data. The number of such cases was:

3

The SPSS results for the mediating effect of power/provocation need

Run MATRIX procedure:

***************** PROCESS Procedure for SPSS Release 2.13 ***************

Written by Andrew F. Hayes, Ph.D. www.afhayes.com

Documentation available in Hayes (2013). www.guilford.com/p/hayes3

**************************************************************************

Model = 4

Y = recom

X = group

M = exist

Sample size

133

**************************************************************************

Outcome: exist

Model Summary

R R-sq MSE F df1 df2 p

.6246 .3901 .9323 83.7919 1.0000 131.0000 .0000

Model

coeff se t p LLCI ULCI

constant 5.4222 .1180 45.9658 .0000 5.1889 5.6556

group -1.5328 .1675 -9.1538 .0000 -1.8641 -1.2016

**************************************************************************

Outcome: recom

Model Summary

R R-sq MSE F df1 df2 p

.9930 .9861 .0225 4614.5795 2.0000 130.0000 .0000

Model

coeff se t p LLCI ULCI

constant 9.1068 .0758 120.0775 .0000 8.9568 9.2569

exist -1.0079 .0136 -74.2592 .0000 -1.0347 -.9810

group .0405 .0333 1.2169 .2258 -.0254 .1064

******************** DIRECT AND INDIRECT EFFECTS *************************

Direct effect of X on Y

Effect SE t p LLCI ULCI

.0405 .0333 1.2169 .2258 -.0254 .1064

Indirect effect of X on Y

Effect Boot SE BootLLCI BootULCI

exist 1.5449 .1737 1.2101 1.8925

******************** ANALYSIS NOTES AND WARNINGS *************************

Number of bootstrap samples for bias corrected bootstrap confidence intervals:

10000

Level of confidence for all confidence intervals in output:

95.00

NOTE: Some cases were deleted due to missing data. The number of such cases was:

3

# Figure 3

social exclusion types

willingness of WOM recommendation recommendation

affiliative-focused needs

Indirect effcet：*β* = 1.94; *CI* = 1.34 to 2.64

Popularity product: β = 1.98; CI = 1.71 to 2.26

Scarcity product: β = 0.04; CI = -0.56 to 0.61

product attributes

social exclusion types

willingness of WOM recommendation recommendation

power/provocation needs

Indirect effcet：*β* = 1.94; *CI* = 1.31 to 2.64

product attributes

Popularity product: β = 1.94; CI = 1.68 to 2.20

Scarcity product: β = 0.003; CI = -0.62 to 0.62

**Mediator = affiliative-focused need**

Run MATRIX procedure:

***************** PROCESS Procedure for SPSS Release 2.13 ***************

Written by Andrew F. Hayes, Ph.D. www.afhayes.com

Documentation available in Hayes (2013). www.guilford.com/p/hayes3

**************************************************************************

Model = 15

Y = recomm

X = exclusi

M = relation

V = product

Sample size

185

**************************************************************************

Outcome: relation

Model Summary

R R-sq MSE F df1 df2 p

.7388 .5458 .7897 219.8985 1.0000 183.0000 .0000

Model

coeff se t p LLCI ULCI

constant 3.6491 .0932 39.1733 .0000 3.4653 3.8329

exclusi 1.9379 .1307 14.8290 .0000 1.6801 2.1957

**************************************************************************

Outcome: recomm

Model Summary

R R-sq MSE F df1 df2 p

.8480 .7192 .4661 91.6751 5.0000 179.0000 .0000

Model

coeff se t p LLCI ULCI

constant 3.4553 .3125 11.0577 .0000 2.8387 4.0719

relation .0207 .0786 .2640 .7920 -.1343 .1758

exclusi -.4050 .2131 -1.9007 .0589 -.8254 .0155

product -3.7472 .4454 -8.4134 .0000 -4.6261 -2.8683

int_1 1.0012 .1158 8.6489 .0000 .7728 1.2297

int_2 .5439 .3006 1.8094 .0721 -.0493 1.1370

Interactions:

int_1 relation X product

int_2 exclusi X product

******************** DIRECT AND INDIRECT EFFECTS *************************

Conditional direct effect(s) of X on Y at values of the moderator(s):

product Effect SE t p LLCI ULCI

.0000 -.4050 .2131 -1.9007 .0589 -.8254 .0155

1.0000 .1389 .2120 .6551 .5133 -.2795 .5572

Conditional indirect effect(s) of X on Y at values of the moderator(s):

Mediator

product Effect Boot SE BootLLCI BootULCI

relation .0000 .0402 .2981 -.5601 .6085

relation 1.0000 1.9805 .1373 1.7133 2.2575

******************** INDEX OF MODERATED MEDIATION ************************

Mediator

Index SE(Boot) BootLLCI BootULCI

relation 1.9403 .3265 1.3393 2.6376

When the moderator is dichotomous, this is a test of equality of the

conditional indirect effects in the two groups.

******************** ANALYSIS NOTES AND WARNINGS *************************

Number of bootstrap samples for bias corrected bootstrap confidence intervals:

10000

Level of confidence for all confidence intervals in output:

95.00

NOTE: Some cases were deleted due to missing data. The number of such cases was:

15

------ END MATRIX -----

**Mediator = power/provocation need**

Run MATRIX procedure:

***************** PROCESS Procedure for SPSS Release 2.13 ***************

Written by Andrew F. Hayes, Ph.D. www.afhayes.com

Documentation available in Hayes (2013). www.guilford.com/p/hayes3

**************************************************************************

Model = 15

Y = recomm

X = exclusi

M = exist

V = product

Sample size

185

**************************************************************************

Outcome: exist

Model Summary

R R-sq MSE F df1 df2 p

.7426 .5514 .8473 224.9192 1.0000 183.0000 .0000

Model

coeff se t p LLCI ULCI

constant 5.5548 .0965 57.5675 .0000 5.3645 5.7452

exclusi -2.0302 .1354 -14.9973 .0000 -2.2972 -1.7631

**************************************************************************

Outcome: recomm

Model Summary

R R-sq MSE F df1 df2 p

.8504 .7231 .4595 93.5038 5.0000 179.0000 .0000

Model

coeff se t p LLCI ULCI

constant 3.5437 .4318 8.2072 .0000 2.6916 4.3957

exist -.0019 .0773 -.0246 .9804 -.1545 .1507

exclusi -.3670 .2119 -1.7316 .0851 -.7852 .0512

product 5.2121 .6277 8.3034 .0000 3.9734 6.4508

int_1 -.9542 .1100 -8.6714 .0000 -1.1713 -.7370

int_2 .4752 .2993 1.5878 .1141 -.1154 1.0658

Interactions:

int_1 exist X product

int_2 exclusi X product

******************** DIRECT AND INDIRECT EFFECTS *************************

Conditional direct effect(s) of X on Y at values of the moderator(s):

product Effect SE t p LLCI ULCI

.0000 -.3670 .2119 -1.7316 .0851 -.7852 .0512

1.0000 .1082 .2113 .5121 .6092 -.3088 .5253

Conditional indirect effect(s) of X on Y at values of the moderator(s):

Mediator

product Effect Boot SE BootLLCI BootULCI

exist .0000 .0039 .3128 -.6182 .6189

exist 1.0000 1.9410 .1303 1.6820 2.1965

******************** INDEX OF MODERATED MEDIATION ************************

Mediator

Index SE(Boot) BootLLCI BootULCI

exist 1.9371 .3387 1.3092 2.6433

When the moderator is dichotomous, this is a test of equality of the

conditional indirect effects in the two groups.

******************** ANALYSIS NOTES AND WARNINGS *************************

Number of bootstrap samples for bias corrected bootstrap confidence intervals:

10000

Level of confidence for all confidence intervals in output:

95.00

NOTE: Some cases were deleted due to missing data. The number of such cases was:

15

------ END MATRIX -----
