# Supplementary material for: The Influence of Social Exclusion Types on Individuals' Willingness to Word-of-Mouth Recommendation
Source: Front Psychol. 2022 Apr 15;13:862003. doi: 10.3389/fpsyg.2022.862003 (PMC9051444; doi:10.3389/fpsyg.2022.862003)
Supplement: Supplementary file 5 [file Data_Sheet_1.pdf]

The appendix

Basic Needs (Williams, 2009)

For each question, please circle the number to the right that best represents the feelings you were experiencing during the game

Not at all Extremely

Belonging

I felt “disconnected” (R)

I felt rejected (R)

I felt like an outsider (R)

I felt I belonged to the group

I felt the other players interacted with me a lot

Self-esteem

I felt good about myself

My self-esteem was high

I felt liked

I felt insecure (R)

I felt satisfied

Meaningful existence

I felt invisible (R)

I felt meaningless (R)

I felt nonexistent (R)

I felt Important

I felt useful

Control

I felt powerful

I felt I had control over the course of the game

I felt I had the ability to significantly alter events

I felt I was unable to Influence the action of others (R)

I felt the other players decided everything (R)

Williams, K. D. (2009). Ostracism: A temporal need-threat model. *Adv. Expe. Soc.Psychol.* 41, 275-314. doi: 10.1016/S0065-2601(08)00406-1

Perceived Degree of Being Ignored and Rejected (Molden et al., 2009)

7-point

Please report the extent to which you felt rejected.

Please report the extent to which you felt neglected.

Emotion state

7-point Semantic Differential Scales (Hagtvedt, 2011)

"not at all happy/very happy"

"not at all excited/very excited"

"not at all hopeful/ very hopeful"

"in a bad mood/in a good mood"

Psychological Distance (Niu et al., 2010)

Instructions

Respondents see seven pairs of circles that range from just touching to almost completely overlapping. One circle in each pair is labeled "self," and the second circle is labeled "other." Respondents choose one of the seven pairs to answer the question, "Which picture best describes your relationship with the others?"

7-point

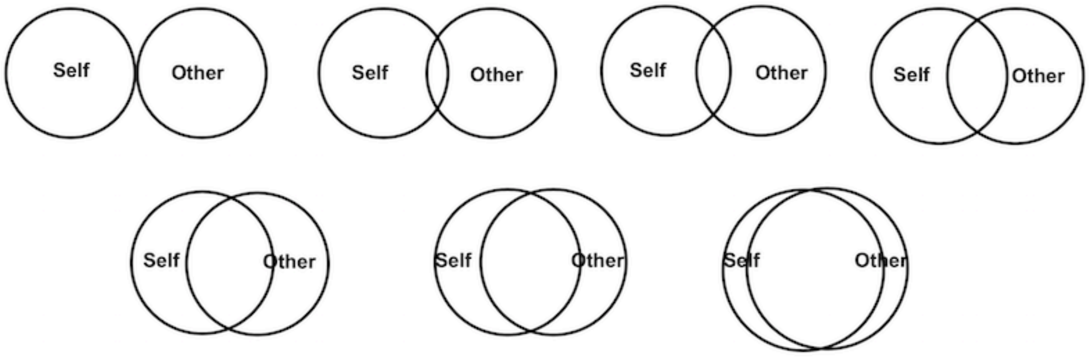

Please report whether your willingness of WOM recommendation is based on the past shopping experience. Yes/No

Please guess the purpose of this activity.
